# Multi-Source Soil Moisture Data Fusion Based on Spherical Cap Harmonic Analysis and Helmert Variance Component Estimation in the Western U.S.

**DOI:** 10.3390/s23198019

**Published:** 2023-09-22

**Authors:** Hao Chen, Peng Chen, Rong Wang, Liangcai Qiu, Fucai Tang, Mingzhu Xiong

**Affiliations:** 1 College of Geomatics, Xi’an University of Science and Technology, Xi’an 710054, China; 21210061019@stu.xust.edu.cn (H.C.); 20210061034@stu.xust.edu.cn (R.W.); 21210061025@stu.xust.edu.cn (L.Q.); 21210226068@stu.xust.edu.cn (F.T.); 21210061029@stu.xust.edu.cn (M.X.); 2 State Key Laboratory of Geodesy and Earth’s Dynamics, Innovation Academy for Precision Measurement Science and Technology, CAS, Wuhan 430077, China; 3 Beijing Key Laboratory of Urban Spatial Information Engineering, Beijing 100045, China

**Keywords:** soil moisture, data fusion, spherical cap harmonic analysis, Helmert variance component estimation

## Abstract

Soil moisture (SM) is a vital climate variable in the interaction process between the Earth’s atmosphere and land. However, global soil moisture products from various satellite missions and land surface models are affected by inherently discontinuous observations and coarse spatial resolution, which limits their application at fine spatial scales. To address this problem, this paper integrates three diverse types of datasets from in situ, satellites, and models through Spherical cap harmonic analysis (SCHA) and Helmert variance component estimation (HVCE) to produce 1 km of spatio-temporally continuous SM products with high accuracy. First, this paper eliminates the bias between different datasets and in situ sites and resamples the datasets before data fusion. Then, multi-source SM data fusion is performed based on the SCHA and HVCE methods. Finally, this paper evaluates the fused products from three aspects, including the performance of representative sites under different climate types, the overall performance of validation sites, and the comparison with other products. The results show that the fused products have better performance than other SM products. In the representative sites, the minimal correlation coefficient (R) of the fused products is above 0.85, and the largest root mean square error (RMSE) is below 0.040 m^3^ m^−3^. For all validation sites, the R and RMSE of the fused products are 0.889 and 0.036 m^3^ m^−3^, respectively, while the R for other products is below 0.75 and the RMSE is above 0.06 m^3^ m^−3^. In comparison to other SM products, the fused products exhibit superior performance, generally align more closely with in situ measurements, and possess the ability to accurately and finely capture the spatial and temporal variability of surface SM.

## 1. Introduction

Surface soil moisture (referred to as SM in the following) is one of the key climatic variables affecting the interaction between atmospheric and terrestrial hydrology [1,2,3]. It profoundly influences agricultural production [4,5], runoff generation [6], drought development [7,8,9], and many other processes. Therefore, timely and accurate information on SM content and its spatial and temporal variability is essential for many applications (e.g., monitoring floods and droughts, irrigation scheduling, improving short- and medium-term meteorological models, and predicting vegetation dynamics) [10]. Over the past decades, SM has been obtained only by in situ measurement, which is spatially under-representative, expensive, and challenging to satisfy the demands of various fields. Consequently, efforts have been devoted to monitoring surface SM through remote sensing techniques such as active and passive microwave sensors.

Several satellite platforms carrying different passive microwave sensors can provide continuous global-scale estimates of SM at the surface (approximately 0–5 cm). The European Space Agency’s [11] Soil Moisture and Ocean Salinity (SMOS), launched on 2 November 2009, is the first mission to provide, from microwave L-band (1.4 GHz, 21 cm) observations, global observations of variability in SM and sea surface salinity [12]. NASA's SMAP was launched on 31 January 2015 and carries both an L-band microwave radiometer and radar, the combination of which improves the retrieval accuracy and spatial resolution of SM [13,14]. In addition, many other in-orbit satellite sensors can monitor surface SM via C- or X-band, including the Microwave Radiation Imager (MWRI) of the Fengyun series and the Trivehe Advanced Microwave Scanning Radiometer (AMSR2) of the Japan Aerospace Exploration Agency (JAXA). Among them, the AMSR2 on board the GCOM-W1 satellite is the successor to the AMSR-E on NASA's satellite Aqua [15,16]. The Cyclone Global Navigation Satellite System (CYGNSS), launched on December 15, 2016, is NASA's first mission to retrieve surface SM using Global Navigation Satellite System (GNSS) signals reflected from the Earth’s surface [17,18,19]. Active microwave sensors include the Advanced Microwave Instrument (AMI) scatterometer [20], the Advanced Scatterometer (ASCAT) of the MetOp series [21], and the CSAR instrument on board the Sentinel-1 satellite [22,23]. Passive microwave systems are suitable for large-scale soil moisture monitoring because passive sensors are less affected by vegetation and soil texture and are more sensitive to soil moisture than active microwave systems [24]. However, due to technical limitations in the development and launch of large antennas, passive soil moisture products typically have a coarse spatial resolution (approximately 25–50 km), which cannot meet the application requirements in fine-scale fields [25]. Compared to passive microwave systems, although active microwave detection can obtain SM products with high spatial resolution, the sensors are susceptible to the influence of vegetation canopy structure, surface roughness, and water content, resulting in lower estimation accuracy of these products [26].

In addition to the above methods, another way to obtain SM estimations is through land surface models. For instance, the European Space Agency Soil Moisture Climate Change Initiative (CCI) project directly fuses SM data from multiple single sensors to produce a global-scale product with a spatial resolution of 0.25° [27]. The ERA5-Land reanalysis datasets developed by the Copernicus Climate Change Service (C3S) of the European Center for Medium Weather Forecasting (ECMWF) can provide hourly SM products at a spatial resolution of approximately 9 km [28]. The Global Land Data Assimilation System (GLDAS) incorporates satellite- and ground-based observations utilizing advanced land surface modeling and data assimilation techniques to generate high-quality global surface meteorological databases [29,30]. The Global Land Evaporation Amsterdam Model (GLEAM) is a set of algorithms that separately estimate the different components of land evaporation. It provides root-zone and surface SM products at a spatial resolution of 0.25° and generates multiple evapotranspiration data [31]. Nevertheless, there are limitations to model-based SM products as each surface model has biases, and product performance varies by region and model [32,33].

Research institutes have released various SM products with different spatial and temporal resolutions, spatial coverage, and inversion algorithms. Still, the limitations of coarse spatial resolution make it difficult to apply these products to more extensive and fine-grained applications in drought monitoring, climate modeling, and agricultural production [25]. Hence, there has been a new research trend to generate SM products with high spatiotemporal resolution by fusing multiple SM data sets. Compared with SM products from a single data source, fused SM products are more reliable and provide a more accurate picture of the spatial and temporal variability of surface SM [24]. In past studies, the spatial resolution of 0.1° has become one of the most common high resolutions in hydrology and agriculture [34]. Various fusion algorithms have been employed to integrate data sources with varying resolutions, ranging from straightforward and analytical methods to more intricate approaches [35,36,37]. For example, Song et al. [38] employed a non-linear enhanced soil moisture downscaling model to improve the spatial resolution of the microwave-based SSM product by utilizing optical remote sensing data. Abowarda et al. [39] adopted a combined method of data fusion and machine learning (random forest model) to downscale remote sensing and model-based surface soil moisture (SSM) output, utilizing high-resolution land surface variables such as NDVI, surface albedo, and surface temperature. Peng et al. [37] utilized the triple collocation method and a least-squares merging scheme to merge different simulated and satellite-based soil moisture products, such as SMAP, Sentinel 1, and SMOS L4, and obtained downscaled soil moisture data with a spatial resolution of 12.5 km. Das et al. [23] used an active-passive downscaling algorithm to fuse SMAP SSM and high-resolution Sentinel-1A/Sentinel1B data and obtained downscaled SSM products with satisfactory performance at 1 and 3 km resolutions. Although satellite-based data fusion can generate large-scale SM maps, current products and downscaling algorithms are unable to produce SM products with high resolution, high accuracy, and spatiotemporal continuity, which are critical for continuous and fine-scale hydrological research and agricultural production [40,41]. In addition, since in-situ measurements are generally considered reliable values and have complementary advantages with satellite products in providing regional or global SM, it is promising to improve the performance of fusion products by integrating in-situ measurements into the SM fusion framework [42,43,44]. Spherical cap harmonic analysis (SCHA) is a regional modeling technique proposed by Haines [45] in the context of research on local geomagnetic field modeling [46]. In contrast to the spherical harmonic analysis (SHA) applied to global modeling [47], SCHA exhibits superior performance in studying the variation of a certain phenomenon on a small area scale, which has been demonstrated in previous studies [48,49]. Helmert variance component estimation (HVCE) can solve the weight ratio by determining the posterior unit weight variance of different types of observations and has been widely used in various fields [50]. This paper is the first application of SCHA and HVCE to the field of SM and adopts this technique to fuse datasets from in situ, satellites, and ERA5-Land into fused SM products.

## 2. Study Area and Data

### 2.1. Study Area

**Figure 1:**
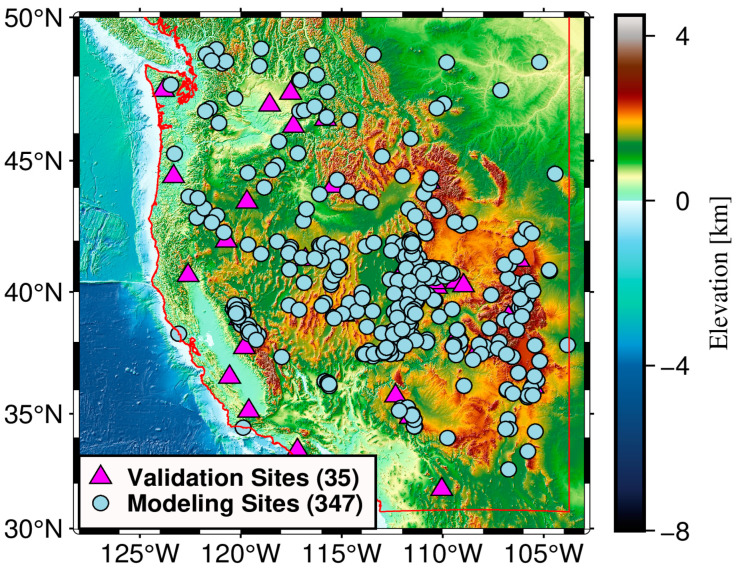
Topography in the study area and distribution of in situ sites from the ISMN (the number in brackets indicates the number of sites, and the red line represents the boundary of the study area).

The study area of this paper is located in the western United States. From the United States Geological Survey (USGS), it can be understood that the western United States has numerous mountain ranges, resulting in elevation changes in the region from below 0 m to 4600 m. The Climate Map of the US provided by United States Maps (https://unitedstatesmaps.org/ (accessed on 23 December 2022)) illustrates that the climate types of the region are broadly classified as Maritime climate, Mediterranean climate, and Alpine climate, and that these different climatic conditions influence the hydrological cycle between the land and the atmosphere. Additionally, various types of land cover (e.g., wetlands, forests, cities, etc.) can also affect the water storage capacity of the soil. With the interaction of the above factors and other external conditions, SM in the study area varies considerably at spatial scales, providing a suitable experimental field for investigating the feasibility of the SCHA and HVCE methods. Furthermore, the in situ sites from the International Soil Moisture Network (ISMN) within the research area are divided into modeling sites and validation sites for constructing the SCHA model and evaluating the fused products, respectively. Figure 1 shows the topography of the study area and the distribution of in situ sites from the ISMN.

### 2.2. Data

The products adopted in this paper can be divided into three specific categories, as follows: (1) Satellite/model-based products, including AMSR2, ERA5-Land, ESA CCI, GLDAS-Noah, GLEAM, MetOp (-A and -B), SMAP, and SMOS; (2) in situ Soil Moisture; (3) auxiliary products, including the Digital Elevation Model (DEM) from SRTM and precipitation from CHIRPS. Considering the co-coverage time of the above products, the research period for this paper is from January 2018 to December 2021. Table 1 summarizes the basic information on the above products, and more details are given in the following subsections.

#### 2.2.1. Satellite/Model-Based Products

AMSR2. The AMSR2 is a remote sensing instrument for detecting weak microwave radiation on the Earth’s surface and in the atmosphere, which can provide climate data [59]. Currently, two types of SM products are published based on AMSR2 data. The first category is released by JAXA, and the second category is based on the Land Parameter Retrieval Model (LPRM) algorithm. This paper adopts the second type of product to avoid radio frequency interference with C-band observations in certain regions, which provides users with a global-scale SM with a spatial resolution of 0.25° in ascending and descending orbits [1,15].

ERA5-Land. The ERA5-Land reanalysis dataset is the fifth generation of atmospheric reanalysis datasets developed by C3S [52,60]. Compared to ERA5 and ERA5-Interim, ERA5-Land is the first global surface variable dataset with a high spatiotemporal resolution that provides SM products that can be classified into four categories [28]. All products have a temporal and spatial resolution of hourly and 0.1°. This paper adopts the first-category (0–7 cm) SM product to ensure the accuracy of the results. For more detailed information on ERA5 Land, please refer to.

ESA CCI. The ESA CCI SM product is derived by merging multiple single sensors and consists of three types of datasets: active, passive, and combined [27], where the combined dataset is the fusion of active and passive datasets [61] and has a higher product accuracy [62]. This paper adopts the combined SM dataset (Version 7.1), released on 21 May 2022, with a spatial resolution of 0.25° and a daily temporal resolution. The new version shows improvements in time and space compared to previous products and is the most accurate ESA CCI SM product.

GLDAS.The Global Land Data Assimilation System (GLDAS) is a land modeling system established by NASA. Advanced land surface modeling and data assimilation techniques are used to integrate satellite- and ground-based observational data products to generate optimal surface state products [29]. At present, GLDAS has published four surface models with a spatial resolution of 0.1° and 0.25°, including Noah [63], the Community Land Model (CLM) [64], the Variable Infiltration Capacity (VIC), MOSIC, and Catchment. This paper selects the SM dataset from Noah with a spatial resolution of 0.25°.

GLEAM. The Global Land Evaporation Amsterdam Model (GLEAM) is a set of algorithms designed to estimate the different components of land evaporation using satellite observational data [31]. GLEAM provides a variety of evaporation products at a global scale with a spatial resolution of 0.25°. In addition, GLEAM also provides surface and root-zone SM. This paper adopts the GLEAM Surface SM (v3.6a) product.

MetOp.ASCAT is an active real aperture radar operating at 5.255 GHz (C-band) and is mainly deployed on the MetOp series (-A, -B, and -C) [65]. This paper adopts the Level 2 SM product with a spatial resolution of 25 km, published by the MetOp series, which provides estimates of the water content of the 0–5 cm topsoil layer, expressed in the degree of saturation between 0 and 100 [%] [66]. Considering that the units of these products are inconsistent with others, this paper first converts saturation (%) into soil volumetric water content (m^3^ m^−3^) through the global soil porosity data provided by ESA CCI.

SMAP. Launched on 31 January 2015, the Soil Moisture Active and Passive (SMAP) is the second satellite for SM monitoring through the L-band after SMOS [67]. This paper adopts the half-orbit SM product with a spatial resolution of 36 km provided by the passive microwave radiometer. Afterward, soil moisture with values exceeding 0–0.6 m^3^ m^−3^ in the dataset was filtered out, and data with the retrieval quality flag set to “recommended retrieval” were screened out [55].

SMOS. Soil Moisture and Ocean Salinity (SMOS), launched by ESA on 2 November 2009, is the first satellite to provide global SM estimates via L-band (1.4 GHz) [12,68]. SMOS operates in the polar orbit and can continuously monitor global surface SM with a revisit cycle of 2–3 days [65]. Currently, SMOS mainly provides the three available surface SM products (SMOS-IC, Level 2, and Level 3). This paper adopts the Level 2 product with a spatial resolution of 15 km, which is freely available to users through the ESA SMOS Online Dissemination Service.

For more information on the above products, please refer to the corresponding references.

#### 2.2.2. In Situ Soil Moisture

The ISMN is an open-source international collaboration to collect and harmonize the maintenance of a global in situ SM dataset [69,70]. The ISMN is capable of providing hourly SM measurements, which are widely applied for evaluating satellite SM products and establishing land surface climate models. The in situ measurements used in this paper are from three monitoring networks; see Table 2 for details. To guarantee the accuracy of the results, 382 sites (35 sites for validation and 347 sites for modeling) are selected based on the following criteria: (i) only sites with a measurement depth of 0–5 cm are considered; (ii) do not select sites with multiple sensors to avoid measurement differences between sensors. Afterward, the observations are filtered according to the quality flags provided by the ISMN, and only those tagged with “G” are retained. Finally, the average of all observations in a day is taken as the SM observation of the site for that day.

#### 2.2.3. Auxiliary Products

Climate Hazards Group InfraRed Precipitation With Station Data (CHIRPS) is a quasi-global rainfall dataset spanning more than 35 years, with a spatial coverage of 50° S–50° N [71]. Studies show that SM changes are strongly correlated with rainfall and topography [72]; therefore, this paper utilizes the bilinear interpolation method to obtain in situ rainfall data from the CHIRPS dataset for the analysis of the results. The DEM is derived from the Shuttle Radar Topography Mission (SRTM) data released by NASA with a resolution of 90 m.

**Figure 2:**
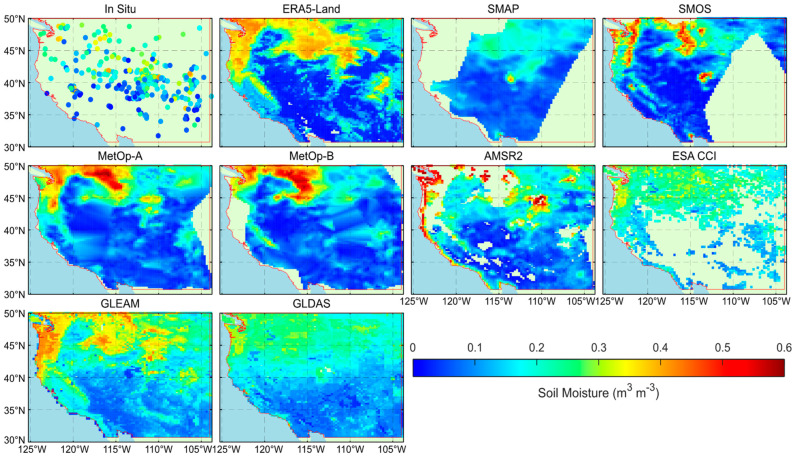
Different SM products used for modeling on 10 May 2020 (the red lines represent the study area boundaries).

Figure 2 shows the SM data and spatial coverage for different products on 10 May 2020. As seen in the figure, the spatial coverage and SM estimates for the study area are distinct for various SM products, which is related to the orbits of the satellites and the data retrieval algorithms. However, the SM estimates are closer for the same series of satellites (e.g., MetOp series), probably because they carry the same sensors and adopt retrieval algorithms with fewer differences.

## 3. Methods and Evaluation Metrics

### 3.1. Spherical Cap Harmonic Analysis

The variables measured on or above the Earth’s surface naturally suit a mathematical description in a spherical coordinate system, of which the most common model is spherical harmonic analysis (SHA) [73]. However, since the associated Legendre function of SHA is only globally orthogonal over the whole sphere, SHA cannot provide well-fitting results in the local area. For these reasons, Haines [45] proposed a technique suitable for spherical regional modeling called spherical cap harmonic analysis (SCHA). Specifically, the SCH functions are constructed by the following steps: First, the spherical harmonic functions are expanded into a set of basis functions. Then, an orthogonal polynomial function is defined on the spherical cap, where orthogonal means that the integral of the polynomial function over the sphere cap is zero. Finally, the SCH functions are obtained by multiplying the spherical harmonic and orthogonal polynomial functions. These SCH functions are orthogonal on the spherical cap and constitute a complete orthogonal basis on the spherical cap.

The critical advantage of modeling with SCHA over SHA is that it allows the use of relatively few SCHA coefficients to represent the data, enables more accurate and efficient processing of data within a specific region of the sphere, and can also better adapt to the distribution characteristics of the data, thus improving the accuracy of data processing. The basic idea of SCH analysis is to express the data on the sphere as a linear combination of a set of SCH functions. At present, SCHA has become one of the preferred region modeling techniques [48], such as regional ionospheric modeling [46,74], geomagnetic field [75,76] and ocean field [77], due to its ability to accurately represent data on the sphere using a small number of coefficients. Additionally, SCH analysis can also be used to filter out the noise and extract relevant features from the data. Overall, the use of SCHA over SHA can provide significant benefits in terms of accuracy and efficiency when processing data in a specific region of the sphere. For more details about the spherical cap harmonic analysis theory, please refer to Haines [45]. This paper is the first application of SCHA to *SM* fields.

The spherical cap harmonic model for mapping the regional *SM* can be expressed as the following equation:

(1) $$SM=\sum_{k=0}^{K_{\max}}\sum_{m=0}^{k}a{\left(\frac{a}{r}\right)}^{n_{k}(m)+1}P_{n_{k}(m)}^{m}(\cos\theta )\cdot \left\{g_{k}^{m}\cos(m\lambda )+h_{k}^{m}\sin(m\lambda )\right\}$$

where, r, θ, λ represents the radial distance, colatitude, and longitude of the point of interest in the spherical coordinate system, respectively. a is the radius of the earth’s surface and equals 6,378,137 m in this paper. $P_{n_{k}(m)}^{m}$ is called the first kind of associated Legendre function, and its solution is obtained by separating the variables and solving a single eigenvalue problem [45]. The parameters $n_{k}(m)$ (non-integer real) and m (integer) represent the degree and order of the SCHA model, respectively, indicating the coupling between the three separated differential equations, and k is an integer subscript of $n_{k}(m)$. $g_{k}^{m}$ and $h_{k}^{m}$ are referred to as the spherical harmonic coefficients to be determined and represent the amplitude of the respective harmonics. $K_{\max}$ is the maximum truncation degree of SCHA, and if the expansion of Equation (1) is truncated at $k=K_{\max}$, the number of coefficients is ${\left(K_{\max}+1\right)}^{2}$ and can determine their values based on the least squares method.

The following problems need to be solved before constructing the SCHA model using SM products: (1) coordinate conversion; (2) determination of the value of $n_{k}(m)$; and (3) calculation of the associated Legendre functions.

**Figure 3:**
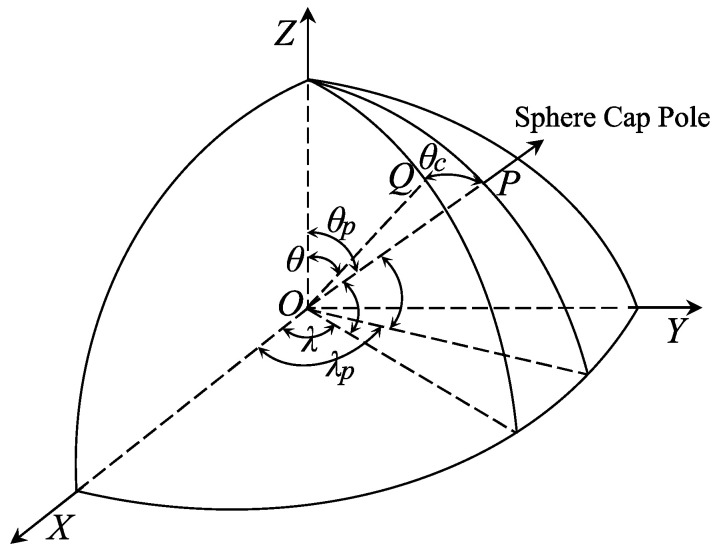
Coordinate transformation between the geographic coordinate system and the spherical cap coordinate system [74].

The coordinate conversion mainly refers to converting the geographical colatitude (θ) and longitude (λ) of the original data from the geographical coordinate system to the spherical cap coordinate system under a new pole. Figure 3 shows the relationship between the geographic coordinate system and the spherical coordinate system [46,74]. Assume that the geographical coordinate of any point Q is (θ, λ) and the coordinate of the pole of the spherical cap is ($\theta_{0}$, $\lambda_{0}$). The coordinate of that point under the spherical cap ($\theta_{c}$, $\lambda_{c}$) can be computed according to the following equations:

(2) $$\left\{\begin{array}{l} \cos\left(\theta_{c}\right)=\cos\left(\theta_{0}\right)\cos(\theta )+\sin\left(\theta_{0}\right)\sin(\theta )\cos\left(\lambda -\lambda_{0}\right)\\ \tan\left(\pi -\lambda_{c}\right)=\frac{\sin(\theta )\sin\left(\lambda -\lambda_{0}\right)}{\sin\left(\lambda_{0}\right)\cos(\theta )-\cos\left(\theta_{0}\right)\sin(\theta )\cos\left(\lambda -\lambda_{0}\right)} \end{array}\right.$$

The Legendre function can be represented as the hypergeometric function [78], which is defined as:

(3) $$F(a,b;c;z)=u=\sum_{k=0}^{\infty }\frac{{\left(a\right)}_{k}{\left(b\right)}_{k}}{k!{\left(c\right)}_{k}}z^{k},\;\;\;\;|z|<1$$

where, ${\left(a\right)}_{k}$, ${\left(b\right)}_{k}$ and ${\left(c\right)}_{k}$ are shifted factorials, defined as:

(4) $${\left(a\right)}_{0}=1,\;\;\;\;{\left(a\right)}_{n}=a(a+1)\ldots (a+n-1),\;\;\;\;n=1,2,\ldots$$

For computing $n_{k}(m)$, we first regard $P_{l}^{m}(t_{0})$ and $P_{l}^{m}(t_{0})/dt$ as functions about l, then calculate the solutions of the following equations separately [45]:

(5) $$\left\{\begin{array}{l} P_{l}^{m}(t_{0})=0\\ \frac{dP_{l}^{m}(t_{0})}{dt}=0 \end{array}\right.$$

The simplified form of Equation (3) can be obtained by conversion [77]:

(6) $$\left\{\begin{array}{l} F(l,m,t_{0})=0\\ lt_{0}F(l,m,t_{0})-(l-m)F(l-1,m,t_{0})=0 \end{array}\right.$$

where, $t_{0}=\cos\theta_{0}$, l denotes $n_{k}(m)$, $F(l,m,t)=F\left(m-l,m+l+1;m+1;\frac{1-t}{2}\right)$, and $\theta_{0}$ is the half angle of the spherical cap, which together with r determines the range of the spherical cap. Then solve Equation (6) for the zeros according to Mueller’s method. When the value of $n_{k}(m)$ is determined, the associated Legendre functions can be computed.

The Schmidt normalized associated Legendre functions $P_{n_{k}(m)}^{m}(\cos\theta )$ (−1<cosθ≤1) in Equation (1) can be expressed when $n_{k}(m)$ is real and m is positive integral real as:

(7) $$\left\{\begin{array}{l} P_{l}^{m}(\cos\theta )=\sum_{k=0}^{\infty }A_{k}(m,l){\left(\frac{1-\cos\theta }{2}\right)}^{k}\\ A_{0}(m,l)=K_{l}^{m}\sin^{m}\theta \\ A_{k}(m,l)=\frac{\left(k+m-1)(k+m)-l(l+1\right)}{k(k+m)}A_{k-1}(m,l) \end{array}\right.$$

where, $K_{l}^{m}$ is the normalizing factor, and its value depends on the kind of normalization.

This paper adopts the Schmidt normalization. The normalizing factor is:

(8) $$\left\{\begin{array}{l} for\;m=0\;\;\;\;K_{l}^{m}=1\\ for\;m\neq 0\;\;\;\;K_{l}^{m}=\frac{2^{-m}}{\sqrt{m\pi }}{\left(\frac{l+m}{l-m}\right)}^{\frac{l}{4}+\frac{1}{4}}P^{\frac{m}{2}}\exp\left(e_{1}+e_{2}+\cdot \cdot \cdot \right) \end{array}\right.$$

where, P, $e_{1}$ and $e_{2}$ can be obtained from the following equations:

(9) $$\left\{\begin{array}{l} P={\left(\frac{l}{m}\right)}^{2}-1\\ e_{1}=-\frac{1}{12m}\left(1+\frac{1}{P}\right)\\ e_{2}=\frac{1}{360m^{3}}\left(1+\frac{3}{P^{2}}+\frac{4}{P^{3}}\right) \end{array}\right.$$

In summary, the preprocessing procedures for *SM* observations in this paper are as follows:

Firstly, the geographical coordinates of observations are converted to spherical cap coordinates, and the center of the study area is usually chosen as the pole of the spherical cap;

Then, calculate $n_{k}(m)$ and $P_{l}^{m}(\cos\theta )$ by the above method, respectively;

Finally, take SM observations, $P_{l}^{m}(\cos\theta )$, and spherical cap coordinates into Equation (1) to obtain linear equations about $g_{k}^{m}$ and $h_{k}^{m}$, which can then be solved by an inversion algorithm such as the least squares method.

### 3.2. Helmert Variance Component Estimation

The estimation of the unknown covariance parameters in the variance matrix is called variance component estimation (VCE), which can simultaneously estimate the variance components and provide a regularization method. In multi-source data fusion, different datasets independent of each other should have distinct weights. However, knowing the relationship between different datasets before data processing is hard, and thus no weights can be determined. Additionally, even though the weights can be determined with existing a priori information, the variation in the number of observations causes the a priori weights not to accurately reflect the relationships between the datasets, while the Helmert variance component estimation (HVCE) method remedies this deficiency by updating the weights during the computation [79,80].

The basic process of HVCE is (1) determine the initial weights of various data sets based on a priori information, which is typically set to equal weights when there is no a priori information; (2) perform parameter estimation (i.e., calculate the unknowns X and residual V) by least squares (or Kalman filter, etc.) method; (3) calculate the unit weight variance of different data sets by corresponding formulas, (4) update the weights of different data sets (i.e., take the variance of one category of data sets as a reference to adjust the weights of other data sets); (5) stop the iteration if the ratio of unit weight variances between different data sets are equal or satisfies the specified conditions, otherwise repeat steps (2)–(4) until the iteration exceeds the given times or satisfies the specified conditions. In this paper, the HVCE is used to carry out the study. The specific calculation steps and equations are given below when there are multiple types of observations.

Assume that a linear system has $n_{t}$ different types of observations; the equations can be listed as follows:

(10) $$\left\{\begin{array}{l} L_{1}=B_{1}X\\ \begin{array}{c} L_{2}=B_{2}X\\ \cdots \end{array}\\ L_{nt}=B_{nt}X \end{array}\right.$$

where, X is a column vector consisting of unknown quantities, $L_{i}\;\;\;(i=1,2,\ldots ,n_{t})$ are $n_{t}$ sets of column vectors representing different observations, and $B_{i}\;\;\;(i=1,2,\ldots ,n_{t})$ are the corresponding coefficient matrices. Since each type of observation has no prior information, it is first assumed that they have the same variance, and the weights of $L_{i}$ is $\lambda_{i}$.

The estimated value can be obtained by the method of least squares and Equation (11):

(11) $$\hat{X}={\left(\lambda_{1}B_{1}^{T}B_{1}+\lambda_{2}B_{2}^{T}B_{2}+\ldots +\lambda_{nt}B_{nt}^{T}B_{nt}\right)}^{-1}\left(\lambda_{1}B_{1}^{T}L_{1}+\lambda_{2}B_{2}^{T}L_{2}+\ldots +\lambda_{nt}B_{nt}^{T}L_{nt}\right)$$

Then, the residuals are calculated according to the following equation:

(12) $$V_{i}=B_{i}X-L_{i}$$

As the formula for HVCE is very complex, matrix inversion is needed after a continuous matrix multiplication; thus, the following approximate equation is often used in the actual calculation:

(13) $${\hat{\sigma }}_{0i}^{2}=\frac{\lambda_{i}V_{i}^{T}V_{i}}{n_{i}}$$

where, ${\hat{\sigma }}_{0i}^{2}$ and $n_{i}$ are the unit weight variance and the number of the *i*-th set of observations, respectively.

The iterative calculation process of the HVCE is as follows:

(a) Estimate the prior weights of different observed values, namely, determine the initial weight value for each type of observation. The initial values of $\lambda_{i}\;\;(i=1,2,\ldots ,n_{t})$ are set to 1 (i.e., equal weights);
(b) Conduct an adjustment for the first time, and obtain $\lambda_{i}V_{i}^{T}V$ according to Equations (11) and (12);
(c) Following Equation (13), obtain the unit weight variance ${\hat{\sigma }}_{0i}^{2}$ of various observations for the first time, and then determine the weights according to the following equation:
  (14) $${\hat{\lambda }}_{i}=\frac{c}{{\hat{\sigma }}_{0i}^{2}}\lambda_{i}$$
  where, *c* is a constant. Generally, one of the ${\hat{\sigma }}_{0i}^{2}$ values is selected.
(d) Steps (b)–(c) are repeated until the unit weight variances ${\hat{\sigma }}_{0i}^{2}$ of various observations are almost equal.

The HVCE can make the unit-weighted variances of different types of observations close through continuous iteration, while estimates of the solution and variance are obtained during each run, which helps to determine the appropriate weights between different observations.

### 3.3. Evaluation Metrics

To evaluate the performance of different SM products, four widely used evaluation metrics are selected to assess the accuracy of the products: correlation coefficient (*R*), root mean square error (*RMSE*), mean absolute error (*MAE*), and mean error (*ME*). The equation is defined as follows:

(15) $$\left\{\begin{array}{l} R=\frac{\mathrm{cov}\left(SM_{i}^{fit},SM_{i}^{real}\right)}{\sigma_{SM_{i}^{fit}}\times \sigma_{SM_{i}^{real}}}\\ RMSE=\sqrt{\frac{1}{N}\sum_{i=1}^{N}{\left(SM_{i}^{fit}-SM_{i}^{real}\right)}^{2}}\\ Bias=\frac{1}{N}\sum_{i=1}^{N}\left(SM_{i}^{fit}-SM_{i}^{real}\right)\\ MAE=\frac{1}{N}\sum_{i=1}^{N}\left|SM_{i}^{fit}-SM_{i}^{real}\right|\\ ubBias=\sqrt{{RMSE}^{2}-{Bias}^{2}} \end{array}\right.$$

where, $SM_{i}^{fit}$ represents the model SM estimates; $SM_{i}^{real}$ represents the in situ *SM* observations; *i* = 1, 2, 3, …, *N*; *N* is the total number of model observations; $\mathrm{cov}(SM_{i}^{fit},SM_{i}^{real})$ is the covariance of $SM_{i}^{fit}$ and $SM_{i}^{real}$; $\sigma_{SM_{i}^{fit}}$ and $\sigma_{SM_{i}^{real}}$ are the standard deviations of $SM_{i}^{fit}$ and $SM_{i}^{real}$, respectively. The evaluation indicators for all products are calculated using the above formulae and used for subsequent comparison of results.

## 4. Results

This paper fuses SM data from satellite/model and partly in situ sites (i.e., modeling sites) by SCHA and HVCE and generates daily fused SM maps. The 35 selected validation sites are not involved in the fusion but are used for product evaluation. Since SM measurements from in situ sites are generally considered authentic values [65,81], this paper eliminates the bias between SM estimates for different products and in situ measurements before performing data fusion to reduce its impact on the accuracy of fused products as much as possible. The specific procedure is as follows:

(a) Expand 0.5° around the in situ sites and calculate the average bias between the SM estimates for the products and the in situ measurements in the area (sites without SM estimates are not involved in the calculation).
(b) Calculate the mean bias for all sites and take it as the daily global bias for the products.
(c) Using the calculated bias to calibrate the SM estimates of the product.

It should be noted that all bias calculations are based on in situ measurements minus the product SM estimates. This paper uses SM from in situ sites and calibrated products to construct an SCHA model of degree 10. The reason for using degree 10 instead of degree 11 is that degree 11 sometimes generates ill-conditioned problems in the calculation process, while degree 10 can avoid this problem.

Since the satellite products adopted in this paper (e.g., SMAP, SMOS) provide SM estimates in the form of ascending and descending orbits, they can only cover part of the study area daily and are, therefore, difficult to apply for accurate product evaluation. Hence, for these reasons, only model-based SM datasets (i.e., ERA5-Land, ESA CCI, GLDAS, and GLEAM) and fused products are used for product evaluation in the following subsections. Additionally, the following calculation results consider only the case where the above SM products are available at the validation sites.

### 4.1. Products Comparison under Various Climate

**Figure 4:**
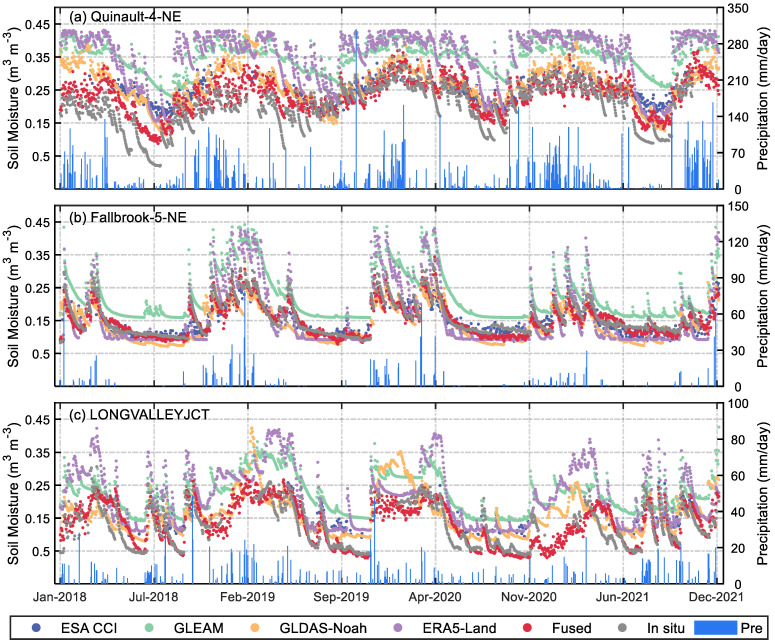
Comparison of SM of different products vs. in situ sites in different climates.

This subsection compares the differences between SM estimates for the fused/model products and in situ measurements under different climatic conditions to evaluate the performance of the fused products. Figure 4 demonstrates the comparison of different products at sites, and the blue bars indicate the daily precipitation. Table 3 presents the calculated results of the evaluation metrics of products at the sites. The comparison results indicate that all products can capture the temporal variation of soil moisture. However, in terms of accuracy, the fused product performs the best, followed by GLDAS-Noah and ESA CCI. Meanwhile, ERA5-Land and GLEAM perform the worst, with their soil moisture estimates being consistently biased towards higher values over the long term. However, the completeness of the time series values from ESA CCI is the poorest.

Figure 4a shows the comparison results of different SM products at the site named Quinault-4-NE, where the site belongs to the maritime climate. The time series in the figure presents a clear seasonality of SM variation at this site, characterized by lower SM in summer and autumn and higher SM in spring and winter. In addition, the fluctuations of in situ SM and precipitation exhibit excellent consistency in their variation over time, and all products in the figure capture their volatility well on the time scale. However, there are significant differences in the performance of these products compared to the in situ measurements, suggesting that they vary in data sources and SM retrieval algorithms. Moreover, it can be seen from Figure 4a that the fused products are the closest to the in situ site in terms of both time variation and numerical estimation of SM. In contrast, other products significantly overestimate the SM of the site, with the most significant variability between GLEAM and ERA5-Land versus the sites, which is also reflected in Table 3. In Table 3, the R and RMSE of the fused products at this site are 0.916 and 0.038 m^3^ m^−3^, respectively, which are the maximum and minimum values among all products, consistent with the performance of the fused products in Figure 4a. Among the other products, though ERA5-Land and GLEAM can reflect the temporal changes of in situ SM, there are significant differences between their estimates and the in situ measurements, with RMSE reaching 0.15 m^3^ m^−3^. The GLDAS performs slightly worse, while the ESA CCI has the smallest R (0.64) and its RMSE (0.064 m^3^ m^−3^) is also relatively large, indicating that it performs the worst at this site.

Figure 4b demonstrates the performance comparison of different products at the site named Fallbrook-5-NE. The climate type of this validation site is the Mediterranean climate, characterized by hot and dry summers and mild and rainy winters, which is consistent with the precipitation variation at this site. The other products, although able to reflect SM changes over time, still suffer from the overestimation of in situ measurements. Figure 4c presents the performance comparison of different products at LONGVALLEYJCT, a site with an Alpine climate. As shown in Figure 4c, the SM estimates of ERA5-Land and GLEAM have the most significant fluctuations over the time series, while the other products perform similarly to the previous sites. Table 3 suggests that the correlation (R) between all products and in situ SM has decreased at this site. Nevertheless, the R (RMSE) of the fused product is still higher (lower) than other products, which indicates that the fused products still exhibit excellent accuracy under this condition.

The phenomenon in Figure 4 shows that changes in soil moisture in different regions are well correlated with rainfall and climate types, and the fusion product is better than other products in both time series and estimation accuracy. In fact, changes in soil moisture can greatly affect the growth of crops and vegetation. Therefore, soil moisture information becomes more important in areas not covered by data. Inferring approximate soil moisture content based on rainfall and climate characteristics can provide timely reference information for agricultural irrigation and water resource management. There are certain differences in the performance of model/satellite-based SM products under different conditions, which may be related to their input data and inversion algorithms, which requires further research.

The above comparison clearly indicates that fused products outperform other products under various climatic conditions, which not only accurately reflects the trend of in situ SM over time but also provides more accurate SM estimates of sites. To further precisely evaluate the performance of fused products, this paper evaluates all SM products using 35 randomly selected in situ sites (i.e., all validation stations) in the following subsections.

### 4.2. Comparison between Products and Validation Sites

This subsection utilizes all validation sites for SM product evaluation. The results indicate that among all the products, the fused product has the best performance, with the highest R value (above 0.8), the lowest RMSE (below 0.04 m^3^ m^−3^), and the smallest bias from the site data. The R values between other products and validation sites are all below 0.75, and the RMSE values are all above 0.05 m^3^ m^−3^, with a large bias from the in situ measurements.

**Figure 5:**
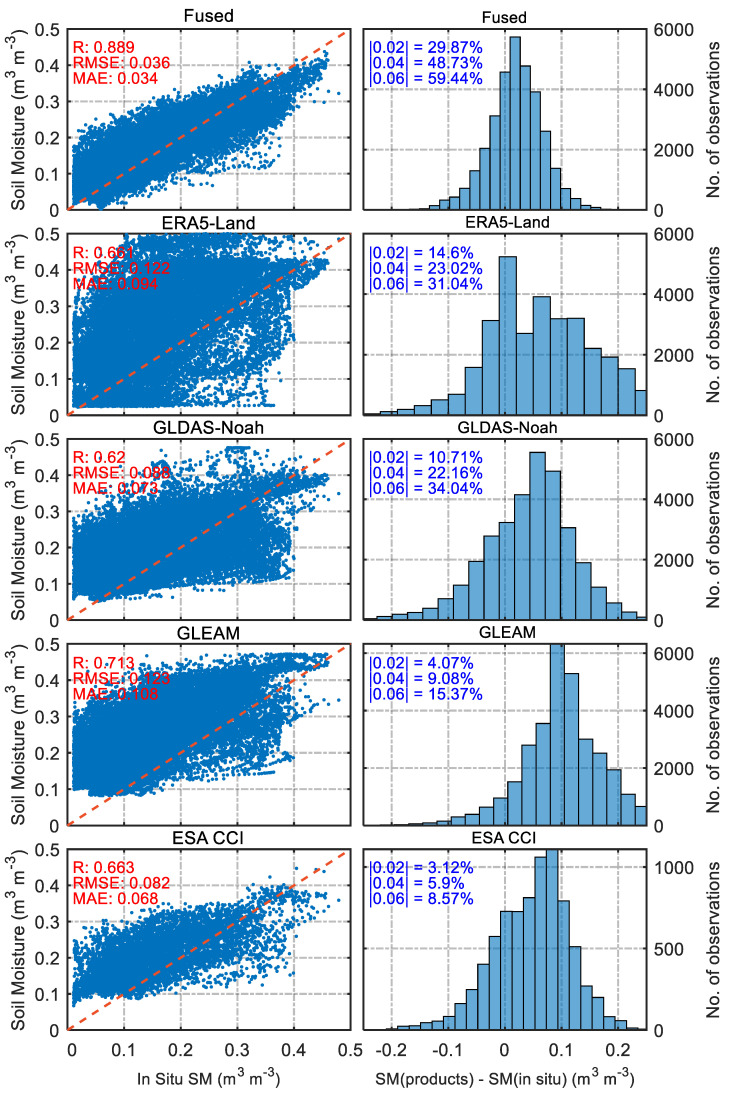
Correlation (**left**) and residual distribution statistical histograms (**right**) between different products and all validation sites.

Figure 5 presents the final comparison results and the statistical histograms of the residual distribution, where |0.02|, |0.04|, and |0.06| represent the probabilities that the absolute values of the residuals are less than or equal to 0.02 m^3^ m^−3^, 0.04 m^3^ m^−3^, and 0.06 m^3^ m^−3^, respectively. Overall, the correlation between the fused products and validation sites reached 0.89, and the percentage of bias less than or equal to 0.04 m^3^ m^−3^ reached 48.73%, which is much higher than the other products, indicating that the fused product also performs well in general. In addition, as seen in the comparative figures and histograms in Figure 5, the SM products of ERA5-Land and GLEAM overestimate the in situ SM in general, which is consistent with the previous conclusions reached at the individual validation site. The correlation between the GLDAS-Noah product and validation sites is 0.620, and the percentage of bias below 0.04 m^3^ m^−3^ is 22.16%. Furthermore, the figures show that the ESA CCI product has a considerably lower amount of data than the other products, indicating that the product covers the study area spatially less well. Again, the SM estimations for this product are slightly higher than the in-situ observations. The above comparison indicates that the fused products are the most accurate of all SM products and can reflect the spatial and temporal variability of in situ SM more realistically and accurately over a large area.

**Figure 6:**
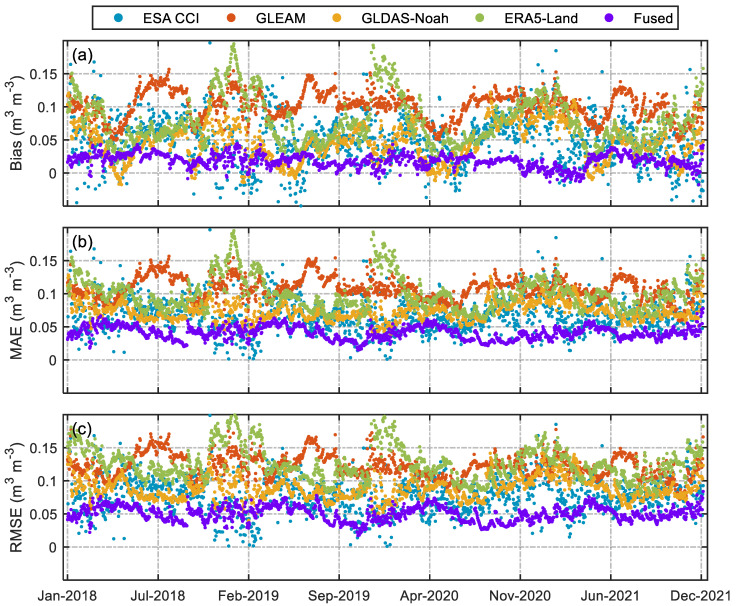
Comparison results of time series of different SM products and validation sites from 2018 to 2021, where (**a**), (**b**), and (**c**) represent Bias, MAE, and RMSE, respectively.

Figure 6 presents the Bias, MAE, and RMSE of different SM products versus in situ SM for 2018–2021. As seen in Figure 6a, the bias of the fused products has the smallest range of variation over the time series, while the ME of the ERA5-Land, GLDAS-Noah, and GLEAM products fluctuates significantly more over the time series than the fused products. The MAE of the fused products is significantly lower than the MAE of the other products in the time series of Figure 6b, which suggests that the fused products are considerably closer to the in situ observations than all the other products. As before, the RMSE of the fused products is the smallest among all products in Figure 6c, and its fluctuation range is minor. The above analysis shows that the fusion product performs the best, while the overestimation of in situ SM by ERA5-Land and GLEAM is obvious.

### 4.3. SM Maps Comparison

**Figure 7:**
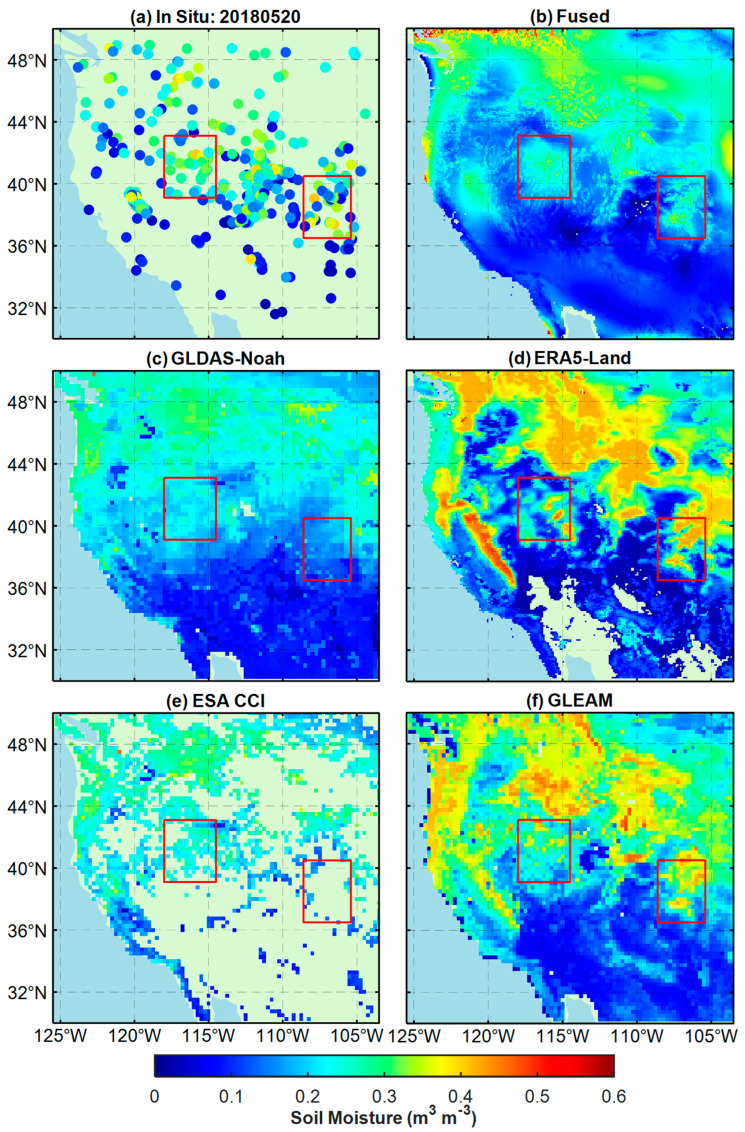
Comparison of SM maps from different types of datasets, including (**a**) In Situ, (**b**) Fused, (**c**) GLDAS-Noah, (**d**) ERA5-Land, (**e**) ESA CCI, and (**f**) GLEAM, on 20 May 2018. Among them, (**a**) represents the measurements; (**b**) represents the fused product of this paper; (**c**,**d**,**f**) represent different model-based products, respectively; and (**e**) represents the fused satellite product. Red rectangular boxes represent areas with significant differences.

**Figure 8:**
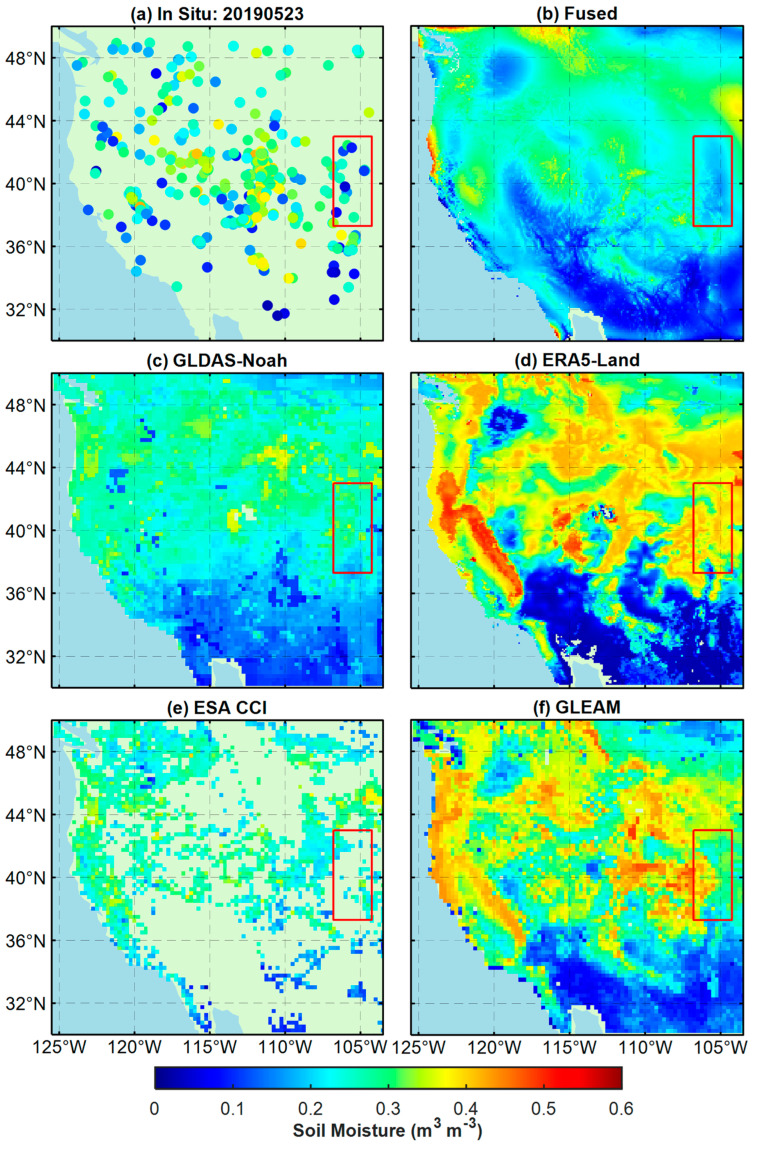
Same as Figure 7, but the date is 23 May 2019 (the red rectangular box represent areas with significant differences).

Except for evaluating the SM products at the validation sites, this paper also compares the in-situ measurements with the SM maps of the five products, as shown in Figure 7 and Figure 8. The results indicate that the fused product can obtain soil moisture information more accurately than other products in local areas and has higher spatial resolution. Compared with in-situ measurements, the fused product is the closest. The soil moisture estimates from ERA5-Land and GLEAM are biased towards higher values, while those from GLDAS-Noah and ESA CCI are biased towards lower values. In addition, the spatial coverage of ESA CCI is the poorest among all the products.

In Figure 7, regarding SM estimates, the fused products in the red rectangle are closest to the in situ, while the ERA5-Land and GLEAM are significantly higher than the in situ. The GLDAS is slightly lower than the in situ Although the ESA CCI product is unable to cover the entire study area, the ESA CCI SM is closer to the in situ SM in the areas where data are available. Regarding spatial changes in SM, the correlation between the fused products and in situ is still the best. The fused SM maps in the red rectangle accurately reflect the variations of in situ SM. The SM maps from ERA5-Land and GLEAM can demonstrate the trend of SM to some extent but are weaker than the fused SM maps in detail, while the SM maps from GLDAS-Noah and ESA CCI cannot reflect the spatial variation of SM well, which implies that both are less correlated with in situ SM. Figure 8 shows that among all the SM products, the fused products perform best in capturing spatial changes and estimating SM. From the fused maps, it can be seen that the soil moisture content in the study area (30° N–37° N) ranged from 0–0.15 m^3^ m^−3^ during this period, indicating that the area is relatively dry and not conducive for vegetation growth, while the soil moisture content in other areas ranged from 0.15–0.35 m^3^ m^−3^, indicating a relatively humid state. The spatial distribution of SM in fused maps shows good agreement with the measurements of the in situ sites. Although the SM maps of ERA5-Land and GLEAM can capture the trend of spatial variation in SM to some extent, their estimated SM values are significantly higher than the measurements at the site. Compared to other SM products, the SM products of GLDAS-Noah and ESA CCI overall underestimated the soil moisture content in the area and cannot accurately characterize the details of SM in some regions (such as in the red rectangular box).

The fused products in Figure 7 and Figure 8 provide spatial information on regional soil moisture at a finer scale, which helps to find areas suitable for planting and study the circulation process of subsurface hydrology. These findings in the results section can bring some practical applications to related research fields.

## 5. Discussion

Comparison results in Section 4 show that the fusion product can estimate surface soil moisture with higher accuracy and spatiotemporal resolution than other products, which is certainly encouraging. In this discussion, this paper further discusses some deficiencies in the research.

**Figure 9:**
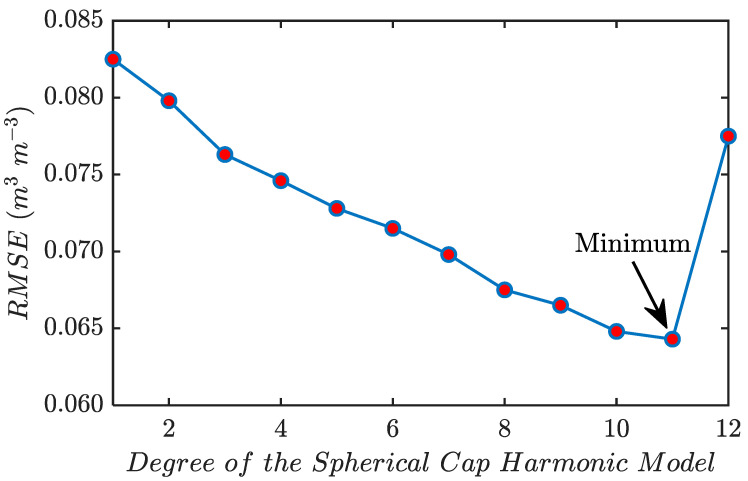
RMSE versus the maximum degree of the SCH model.

To find the best fitting degree and order of the SCH model, a lot of work has been done before multi-source data fusion. Considering the boundary effect of the SCH model, this paper first expands the boundary of the study area appropriately by 2°, where the spherical cap pole is located at 40.922° N, 113.378° W, and the spherical cap half-angle is 15°. The computation results $n_{k}(m)$(0≤k≤12) are shown in Table 4. Then, this paper initializes the model starting from degree 1, gradually increasing its degree, and stops growing the degree when a problem occurs during the calculation. It should be noted that in this study, the maximum order is always equal to the maximum degree. Finally, the accuracy of the model fit is evaluated by calculating the RMSE of each fit. The experimental results showed that the RMSE of the daily fit results showed the same trend as the degree. Figure 9 shows the RMSE versus degree, which indicates that the RMSE of the model gradually decreases from degree 1 to degree 11 and then starts to increase again, which implies that the SCH model obtains the best fitting accuracy at degree 11. The RMSE starts to increase after degree 11 due to the ill-conditioned problem of the matrix in the calculation process, where the solutions of the equations become inaccurate and lead to a large bias between the fitted and true values. Therefore, this article determines that the optimal degree and order of the SCH model are both 10.

Furthermore, since the accuracy of in situ measurements is higher than that of other products, their weight is artificially set to 100 in this paper to ensure they can play a crucial role in data fusion. Afterward, this paper takes the SM dataset of SMAP as the reference (i.e., the weight is constantly equal to 1) and adaptively determines the relative weight relationships of the remaining SM datasets by HVCE. Table 5 gives the weight relationships of SM data from different satellites and models relative to the SMAP dataset during the modeling process. From the table, it can be observed that there are differences in the weights of different data sources because the weight variations are related to the orbits of the satellites and the number of observations. In contrast, the weights of the same series of satellites (e.g., MetOp-A and MetOp-B) vary more similarly.

Whether in the soil moisture field or other fields of multi-source data fusion, using different fusion methods usually results in fusion products with differences in both temporal-spatial resolution and accuracy, which is inevitable. Therefore, more in-depth research is needed to develop soil moisture products with better performance. In further research based on this method, it is necessary to consider how to improve the degree and order of the SCH model based on avoiding the ill-conditioned problem in the calculation process to obtain the soil moisture product with higher spatial resolution. In addition, HVCE is generally suitable for determining weights between datasets with small differences in data volume. However, when there are significant differences in data volume between multiple datasets, it is necessary to consider better methods for determining weights. In the data fusion process, we artificially set the weight of in-situ data to 100, which may not be the optimal solution. Therefore, further research is needed on how to obtain the optimal weight for each type of dataset in the fusion process.

When a study involves a variety of data sets from multiple sources, it is particularly significant to analyze and address the potential bias or error source in the data sources, which is also a crucial step in data fusion. Compared with the SM estimations based on satellite/model, the in situ measurements are undoubtedly closer to the actual soil moisture content; therefore, the in situ measurements are used as the reference data to eliminate the potential bias in the different data sources in this study. However, when there is a lack of in-situ measurements in the study area, this method is obviously ineffective. For this reason, in future research, we need to consider developing more suitable and robust methods that can eliminate potential biases between different data sources. This method is not only limited to this field but is also applicable to other research fields involving multiple datasets. In addition, all the soil moisture data products used in this article are passive remote sensing products. However, active microwave remote sensing, such as Sentinel 1 C band, can also provide soil moisture data. Therefore, in future research, it is possible to consider combining active microwave products to generate higher spatiotemporal resolution fusion products.

## 6. Conclusions

High-quality soil moisture products are essential for hydrological and drought studies and other earth science studies (e.g., water management and climate change). Currently, soil moisture products at large spatial scales are available from satellite retrievals (e.g., SMAP, SMOS), land surface models (e.g., GLEAM), or reanalysis datasets (e.g., ERA5-Land), yet some discrepancies exist between the products from these single data sources and in situ measurements. Therefore, this paper employs SCHA and HVCE to fuse soil moisture datasets from in situ, remote sensing satellites, and ERA5-Land and generate daily fused products in the western United States.

This paper evaluates different soil moisture products in terms of climate type, in situ site validation, and inter-product comparison. The results indicate that: (1) All products can capture the trend of soil moisture in time series at in situ sites under different climatic conditions; however, there are still significant biases between product estimates and in situ measurements. (2) Among all SM products, the R and RMSE between the fusion products and the validation sites are the largest and the smallest, respectively, which means that the precision of the fusion products is the best. This paper also finds that SM estimates from land surface model products and reanalysis datasets tend to be larger than in situ measurements within the study area. (3) Compared with other products, the fusion products can provide closer estimates of surface soil moisture and can reflect the variation of surface soil moisture on a spatial scale in greater detail. It should be noted that the bias reduction is mainly due to prior correction before fusion rather than data fusion.

The fusion method proposed in this paper improves product precision through two main steps. First, the average bias between SM estimates for different products and in situ measurements is calculated separately before data fusion, and it is used to correct the corresponding product data. Second, since in situ measurements are typically considered to have higher observed accuracy, this paper artificially assigns larger weights in constructing the SCHA model and adaptively determines the optimal relative weights among the remaining data sets by HVCE. The above steps ensure that the in situ SM measurements can play a crucial role in data fusion, which is also an essential step in improving the accuracy of the product. The fused products obtained after data fusion are essentially weighted averages of different data sets, which can fully capture the valid information of multiple data sources. In addition, the fusion method proposed in this paper is also applicable to the study of datasets in other partial regions.

## Figures and Tables

**Table 1:** The detailed information on the products used in this paper.

Products (Acronym)	Sensor(s)	Spatial Resolution	Temporal Resolution	Temporal Coverage	Units	Reference(s)
Satellite/Model products						
AMSR2(LPRM) L3	AMSR2	0.25°	Daily	2012–present	percent (%)	[51]
ERA5-Land	Mult-sensors	0.1°	Hourly	1979–present	m^3^ m^−3^	[52]
ESA CCI combined v07.1	Mult-sensors	0.25°	Daily	1978–2021	m^3^ m^−3^	[27]
GLDAS-Noah v2.1	Mult- sensors	0.25°	3-hourly	2000–2022.7	kg m^−2^	[53]
GLEAM v3.6a	Data set	0.25°	Daily	1980–2021	m^3^ m^−3^	[54]
MetOp(-A, -B) L2	ASCAT	~25 km	1–2 days	2007–present	percent (%)	[20]
SMAP L2	Radiometer	~36 km	1–2 days	2015–present	cm^3^ cm^−3^	[55,56]
SMOS L2	Radiometer	15 km	1–2 days	2010–present	m^3^ m^−3^	[12]
Auxiliary products						
CHIRPS v2.0	Data set	0.25°	Daily	1981–present	mm/day	[57]
SRTM DEM	SRTM	~90 m	Multi-Day	Feb 2000	m	[58]

**Table 2:** List of in situ sites from the ISMN adopted (only 0–5 cm depth was used) in this study.

Network Name	No. of Site	Probes Depth (cm)	IGBP Land Cover ^a^	References
SCAN	48	5, 10, 20, 51, 102	Diverse land cover	http://www.wcc.nrcs.usda.gov/ (accessed on 20 August 2022)
SNOTEL	321	5, 10, 20, 51, 102	Diverse land cover	http://www.wcc.nrcs.usda.gov/ (accessed on 20 August 2022)
USCRN	32	5, 10, 20, 50, 100	Diverse land cover	http://www.ncdc.noaa.gov/crn/ (accessed on 20 August 2022)

^a^ International Geosphere-Biosphere Program.

**Table 3:** The calculated results of the evaluation metrics of different products on the related sites.

Products	Quinault-4-NE				Fallbrook-5-NE				LONGVALLEYJCT			
	R	RMSE	ubRMSE	Bias	R	RMSE	ubRMSE	Bias	R	RMSE	ubRMSE	Bias
Fused	0.916	0.038	0.029	0.029	0.952	0.018	0.016	−0.008	0.863	0.035	0.035	0.004
ERA5-Land	0.834	0.155	0.045	0.148	0.920	0.516	0.051	0.009	0.842	0.097	0.051	0.083
ESA CCI	0.644	0.081	0.053	0.062	0.878	0.026	0.024	−0.009	0.853	0.048	0.031	0.036
GLDAS-Noah	0.712	0.076	0.048	0.058	0.809	0.039	0.032	−0.020	0.731	0.050	0.044	0.023
GLEAM	0.771	0.156	0.043	0.149	0.904	0.075	0.035	0.070	0.832	0.092	0.036	0.086

**Table 4:** Value of $n_{k}(m)$ for $\theta_{0}=15{}^{\circ}$.

k	0	1	2	3	4	5	6	7	8	9	10	11	12
0	0												
1	8.68	6.58											
2	14.14	14.14	11.25										
3	20.58	19.88	19.15	15.66									
4	26.30	26.30	25.15	23.93	19.96								
5	32.55	32.12	31.67	30.17	28.58	24.19							
6	38.36	38.36	37.60	36.82	35.04	33.13	28.38						
7	44.54	44.22	43.90	42.88	41.83	39.79	37.61	32.53					
8	50.40	50.40	49.82	49.24	48.00	46.72	44.46	42.04	36.66				
9	56.53	56.28	56.03	55.24	54.45	53.01	51.52	49.07	46.43	40.76			
10	62.42	62.42	61.96	61.49	60.52	59.54	57.93	56.26	53.62	50.78	44.85		
11	68.53	68.32	68.11	67.47	66.83	65.70	64.54	62.77	60.93	58.13	55.09	48.92	
12	74.16	74.47	74.07	73.66	72.85	72.06	70.78	69.47	67.55	65.56	62.60	59.39	52.98

**Table 5:** Weight relationships of SM data from different data sources relative to the SMAP dataset.

Satellite	Min Weight	Max Weight	Mean Weight
SMAP	1	1	1
AMSR2	0.075	0.654	0.239
ERA5-Land	0.082	0.632	0.290
ESA CCI	0.102	1.707	0.832
GLDAS	0.034	1.521	0.644
GLEAM	0.042	0.710	0.289
MetOp-A	0.051	0.690	0.330
MetOp-B	0.050	0.713	0.338
SMOS	0.041	0.580	0.301

## Data Availability

Not applicable.
